# Resting-State Electroencephalogram Depression Diagnosis Based on Traditional Machine Learning and Deep Learning: A Comparative Analysis

**DOI:** 10.3390/s24216815

**Published:** 2024-10-23

**Authors:** Haijun Lin, Jing Fang, Junpeng Zhang, Xuhui Zhang, Weiying Piao, Yukun Liu

**Affiliations:** Heilongjiang Province Key Laboratory of Laser Spectroscopy Technology and Application, Harbin University of Science and Technology, Harbin 150080, China; 2220600029@stu.hrbust.edu.cn (J.Z.); zhangxuhui@hrbust.edu.cn (X.Z.); pwying@163.com (W.P.); liuyukun@hrbust.edu.cn (Y.L.)

**Keywords:** resting-state electroencephalography, major depressive disorder, traditional machine learning, deep learning, artificial intelligence

## Abstract

The global prevalence of Major Depressive Disorder (MDD) is increasing at an alarming rate, underscoring the urgent need for timely and accurate diagnoses to facilitate effective interventions and treatments. Electroencephalography remains a widely used neuroimaging technique in psychiatry, due to its non-invasive nature and cost-effectiveness. With the rise of computational psychiatry, the integration of EEG with artificial intelligence has yielded remarkable results in diagnosing depression. This review offers a comparative analysis of two predominant methodologies in research: traditional machine learning and deep learning methods. Furthermore, this review addresses key challenges in current research and suggests potential solutions. These insights aim to enhance diagnostic accuracy for depression and also foster further development in the area of computational psychiatry.

## 1. Introduction

Depression, a prevalent mental disorder, is characterized by a persistent low mood, anhedonia, or diminished interest in activities, and in severe cases, may lead to suicide [1]. According to the World Health Organization (WHO), an estimated 280 million people worldwide are affected by depression [2,3,4]. Consequently, depression is prioritized in the World Health Organization’s Mental Health Gap Action Programme [5]. Clinically, the diagnosis of a depressive episode typically requires a duration of at least two weeks, accompanied by significant distress or impairment in social functioning. The primary diagnostic criteria for depression are detailed in the Diagnostic and Statistical Manual of Mental Disorders, Fifth Edition (DSM-5) or Fourth Edition (DSM-IV) [6,7]. Diagnoses typically rely on psychiatric evaluations and psychometric questionnaires, including the Hamilton Depression Scale (HAM-D) and the Self-Rating Depression Scale [8,9,10,11,12,13]. However, this approach relies heavily on patient cooperation and clinician expertise, which complicates the diagnostic process and may delay early intervention for patients [14,15]. Depression disrupts neural communication and neurogenesis, impairing the function of specific brain regions and leading to fluctuating patterns of brain activity [16,17]. Research shows that individuals with depression exhibit dynamic disturbances in the frontal limbic network and abnormal inter-cerebral functional connectivity [18,19], which is indicative of altered complex neuronal interactions. Consequently, numerous clinicians and researchers recognize that identifying objective physiological indicators for the direct diagnosis of depression could substantially enhance both diagnostic accuracy and treatment outcomes.

Electroencephalography (EEG) is a non-invasive neuroimaging technique offering a high temporal resolution and easy accessibility [20,21]. EEG accurately reflects brain network activity, offers objective neural markers, and remains free from clinical bias and subjective self-assessment. It has also proved valuable for capturing information about physiological changes in the brain associated with depression [22,23,24]. Compared to magnetic resonance imaging (MRI), EEG is more cost-effective and suitable for frequent testing, thereby offering significant advantages in diagnosing depression [25,26]. Compared to EEG during task execution, resting-state EEG (rsEEG) not only minimizes interference from visual scenes, instructions, and task performance variations influenced by subjects’ cognitive levels, gender, age, and interest [27], but it also effectively captures the intrinsic brain activity. The resting state allows subjects to focus their attention inward, thus facilitating self-related brain activity [28,29]. Furthermore, a study comparing resting-state and task-state EEG demonstrates that classification accuracy is higher in the resting state than in the task state [30]. With advances in computational psychiatry [31,32,33], the integration of rsEEG with artificial intelligence produces remarkable results in diagnosing depression [34]. In contemporary research on depression diagnosis, computer-aided diagnostic models primarily fall into two categories: traditional machine learning (TML) models and deep learning (DL) models. Traditional machine learning models rely on feature engineering to process and analyze training data, using algorithms to make decisions. Conversely, deep learning models can handle large datasets and autonomously learn complex feature representations.

Currently, there are only a few review articles that address the application of EEG in diagnosing depression. Numerous studies highlight the impact of feature extraction and the selection of classification models on diagnostic outcomes; however, few directly compare traditional machine learning methods with deep learning approaches. We compare and analyze common elements such as sample size, data acquisition, preprocessing, feature extraction, feature selection, classification methods, and validation techniques across these two types of studies. Furthermore, we examine the primary challenges in the current research and propose desirable practices. In all tables presented in this review, a dash (“-”) indicates that relevant data are unavailable in the reference.

## 2. Methods

### 2.1. Search Strategy

The use of deep learning methods for diagnosing depression has seen a rapid increase since 2018. Consequently, our study reviews diagnostic research published from 2018 to 2024. The search database used in this review is Web of Science. For the search of the literature, we use the following subject terms: (“Machine learning” OR “Deep learning”) AND (“EEG” OR “Electroencephalography”) AND (“Depression” OR “MDD”). A total of 371 papers are retrieved from the search.

### 2.2. Inclusion Criteria

Initially, the titles and abstracts of all papers are reviewed to exclude duplicates, withdrawn studies, and conference proceedings. Subsequently, the screening process continues with the following criteria:

1. The studies primarily focus on diagnosing depression;
2. The sample comprises both patients diagnosed with MDD and healthy controls (HC);
3. The EEG data consist exclusively of resting-state recordings;
4. Depression is diagnosed using either TML or DL;
5. The study involves tasks related to EEG data acquisition, EEG signal processing, classification, and validation.

The final sample for the comparative analysis comprises 49 relevant articles. Each article employs various signal processing, classification, and validation methods, essential for analyzing and comparing the performance of TML and DL methods.

## 3. Results

**Figure 1:**
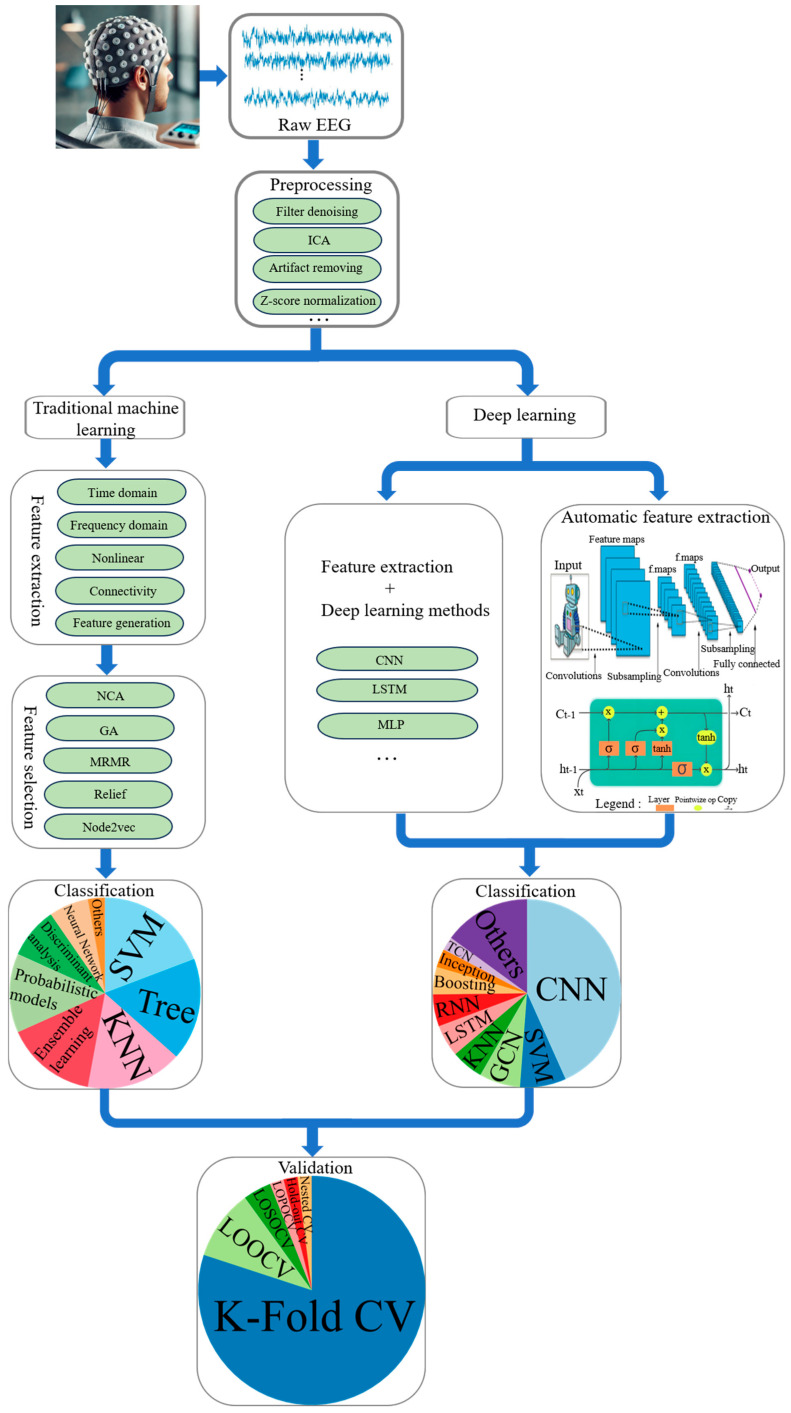
General framework for depression diagnosis based on TML and DL methods.

Forty-nine studies employing rsEEG for depression diagnosis, published between 2018 and 2024, are systematically reviewed. This includes 22 studies employing TML methods and 27 studies utilizing DL approaches. Two of these studies employ both methods [35,36]; they are classified based on the method that achieves the highest accuracy rate in the analysis. Section 3.1, Section 3.2, Section 3.3, Section 3.4, Section 3.5, Section 3.6 and Section 3.7 provide a detailed account of the sample size, data acquisition, and preprocessing approaches used in these studies, along with the methods applied for feature extraction and selection, classification, and validation. Figure 1 illustrates the general framework for diagnosing depression using TML and DL methods. The figure clearly shows that the raw EEG obtained from the EEG cap must first undergo preprocessing, as indicated by the elliptical area in the preprocessing box grid, which highlights the four most commonly utilized methods. Subsequently, two approaches are presented: on the left, the TML method and on the right, the DL method. In TML methods, feature extraction and selection are two distinct steps, with the most frequently used methods detailed in these modules. If a deep learning approach is employed, the feature extraction step includes two strategies: manual feature extraction integrated with a DL model, as depicted in the ellipse area, and automatic feature extraction, with schematic diagrams of the two predominant structures, convolutional neural network and long short-term memory, systematically arranged from top to bottom within the box frame. In the classification and validation module, each segment’s size in the sector diagram corresponds to the frequency of use of each method.

### 3.1. Sample Size

#### 3.1.1. Studies Based on TML Methods

In 2012, Ahmadlou and his team conducted the first study on diagnosing depression using traditional machine learning with rsEEG, involving a limited sample size of 24 subjects [37]. In 2018, Cai et al. [38] expanded their study’s sample size to include 213 participants, comprising 92 individuals with depression and 121 controls without depression. Subsequently, Mumtaz et al. [39] published a study including 34 with MDD and 30 age-matched HC. Wan et al. [40] used two separate samples: one consists of 35 participants (23 with MDD and 12 HC), and the other comprises 30 participants (15 with MDD and 15 HC). In 2021, Wu et al. [41] significantly increased their sample size by collecting rsEEG data from 400 participants, evenly divided into 200 with MDD and 200 HC, across four different hospitals. In contrast, in 2022, Avots et al. [42] conducted a study featuring a notably limited sample size of 20 participants, consisting of 10 individuals with MDD and 10 HC. In the same year, Li et al. [43] analyzed data from 92 cases. Two studies by Soni et al. [44,45] used multiple datasets, each with sample sizes ranging from 30 to 55 and incorporating data from the study by Seal et al. [46], which includes 15 patients with MDD and 18 HC. According to the WHO, depression proves more prevalent in women than in men [3]. In 2023, Shim et al. [47] analyzed EEG data exclusively from female participants, consisting of 49 patients with MDD and 49 HC. This study is unique, as it exclusively includes female subjects. In the same year, two other studies included samples of 32 participants (19 with MDD and 13 HC) and 80 participants (40 with MDD and 40 HC), respectively [48,49]. Table 1 outlines the fundamental experimental setup for EEG data acquisition in studies employing both TML and DL methodologies. It features four columns, detailing from left to right: sample size, frequency (in Hz), number of electrodes, and the corresponding study. Table 2 provides the web address of the publicly available dataset used in the depression diagnostic study, which is accessible by clicking on the link. Additionally, numerous researchers have utilized these publicly available sample data in their studies, as detailed in Table 2. The dataset from Mumtaz et al. [39,50,51], which includes 34 patients with MDD and 30 HC, is frequently used by researchers for analysis [52,53,54,55,56,57,58]. Nassib et al. [59] used a public dataset (42 with MDD + 42 HC) published by Cavanagh’s team [60]. The Multi-modal Open Dataset for Mental-disorder Analysis (MODMA), provided by Cai’s team [61] and comprising two subsets—one with 24 patients with MDD and 29 HC, and another with 26 with MDD and 29 HC—significantly contributes to depression detection research conducted by various researchers [44,45,62,63].

#### 3.1.2. Studies Based on DL Methods

In 2018, Acharya et al. [64] published a study on depression detection, collecting EEG signals from 30 participants, comprising 15 with MDD and 15 HC. Subsequently, Mao et al. [81] and Ay et al. [66] published studies with sample sizes of 34 participants (17 with MDD and 17 HC) and 30 participants (15 with MDD and 15 HC), respectively. In 2020, Wan’s team [72] and Duan et al. [35] chose the same data source for their experiments, though their sample sizes differ: Wan et al. included 35 participants (12 with MDD and 23 HC), while Duan et al. analyzed data from 32 participants (16 with MDD and 16 HC). In 2021, Seal et al. [65] analyzed a sample of 33 participants, including 15 with MDD and 18 HC. The samples employed by the researchers mentioned above are relatively small, with none exceeding 35 participants. Subsequently, Khan et al. [79] increased the sample size to 60 participants for depression diagnosis research, dividing them equally between patients with MDD and HC. In 2022, Yan et al. analyzed a dataset of 80 participants, evenly split between 40 with MDD and 40 HC [69,70]. In 2023, Zhang et al. [75] published a study including 53 participants, consisting of 24 with MDD and 29 HC. Subsequently, Xu et al. [73] further increased the sample size to 75 participants, including 41 with MDD and 34 HC, in their analysis. To better validate the proposed methods, an increasing number of researchers are opting to use multiple datasets. In these studies, the sample sizes generally hover around 105 participants, with one study exceeding 200 participants (refer to Table 2). The use of an entirely different set of EEG data renders the results significantly more compelling. Researchers extensively use publicly available datasets in studies employing deep learning frameworks. As indicated in Table 2, the publicly available dataset from Mumtaz’s team [39,50,51,89] is utilized by more researchers than any other, followed by the MODMA dataset from Cai’s team [61]. Furthermore, the datasets from Cavanagh’s team [60], the EDRA dataset shared by Yang et al. [90], and the PRED + CT dataset are also popular among various researchers.

#### 3.1.3. Analysis

As indicated in Table 1, only four studies feature sample sizes exceeding 200, with one study comprising a sample of 400. Nine studies include an equal number of participants in the depression and control groups. Publicly available datasets are used more frequently in research utilizing deep learning frameworks, rather than in research employing traditional machine learning methods (refer to Table 2). Additionally, variations in the samples used across different studies were observed. Table 3 offers a detailed summary of the unpublished datasets used in depression diagnostic studies, including the male-to-female ratio, subjects’ mean age or age range, diagnostic criteria for MDD, data collection sources, and associated studies. As shown in Table 3, the diagnostic methods used in various studies to ascertain the status of MDD patients vary. These methods include the DSM-IV, HAM-D, International Classification of Diseases (ICD), Emotional State Questionnaire (EST-Q), Mini-international Neuropsychiatric Interview (MINI), and Patient Health Questionnaire-9 (PHQ-9), among others [11,91,92,93,94,95,96,97]. Similarly, the state of the participant during the experiment—whether their eyes are open, closed, or both—can significantly influence the EEG signals captured. Additional parameters influencing brain activity include age, sex, the time of day the experiment is conducted, and prior physical activity. Painkillers, antidepressants, or any other medications that significantly alter brain activity can influence the outcomes of the experiments. Similarly, studies vary in terms of participants’ medication use: some require all participants to have ceased any medication use at least six weeks before the experiment; others enroll participants who are receiving medication for the first time; and some include participants who are already on medication. Some studies fail to report participants’ medication use. When selecting subjects, it is advisable to include individuals who are at least 18 years old and have a minimum education level of middle school. Subjects with MDD must meet both DSM-IV and criteria, whereas healthy controls must have no history of mental disorders. Common exclusion criteria for participation include the following: (1) severe physical disabilities that preclude completion of the experiment; (2) diagnoses of other mental or neurological disorders; (3) histories of brain injuries that have resulted in coma; and (4) consumption of alcohol, nicotine, or caffeine prior to the experiment.

### 3.2. Data Acquisition

The process of data acquisition represents the initial stage of EEG research, and data collection methods vary across studies. Next, the included studies are compared based on parameters such as the number of electrodes, sampling rate, and other relevant factors. Initially, Ahmadlou’s team [37] use 19 electrodes to record rsEEG signals from both depressed patients and healthy controls with their eyes closed for three minutes, and the sampling frequency is 256 Hz. Numerous subsequent investigations adopt identical electrode quantities and sampling frequencies, with only slight deviations observed in the sampling duration. As EEG acquisition techniques evolve, a growing number of researchers enhance both channel count and sampling rates to acquire more granular data on rsEEG signals [41,47,49,59]. Yan et al. [69,70] utilize sampling rates of up to 1000 Hz to record EEG signals in their experiments.

Research has shown that effective classification tasks can be accomplished using fewer electrodes [68]. This reduction conserves not only time and computational resources, but it also decreases susceptibility to noise. Fewer electrodes mean a simpler process, which will facilitate the expansion of EEG datasets in this field. Given the strong correlation between the frontal lobe and emotional processes [99], Cai et al. [38] employ a three-electrode frontal system to collect 90 s of data, using the sampling frequency of 250 Hz. Sakib et al. [48] record data from only 14 channels using a wireless EEG headset. Some research teams employ multiple sampling rates for various signal processing methods during experiments to enhance data reliability [40,42].

**Figure 2:**
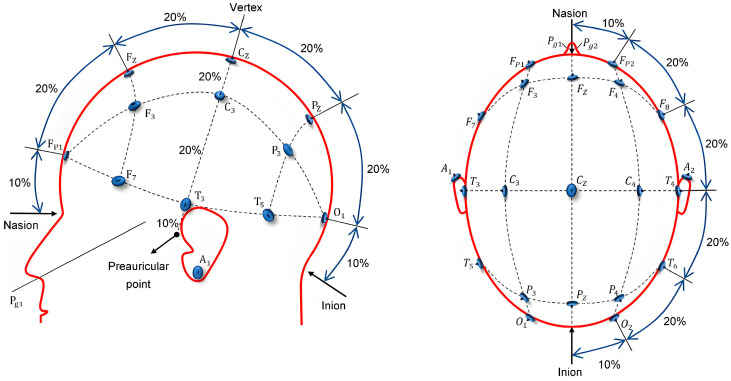
The 10–20 system electrode position diagram, taken from Ref. [68].

**Figure 3:**
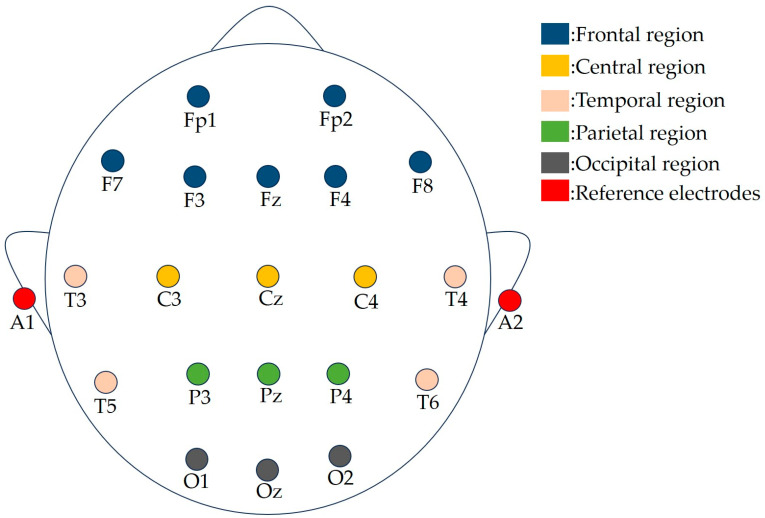
Schematic diagram of brain regions.

Additionally, the placement of the electrodes is of equal importance. The 10–20 system for electrode placement remains the standard method prescribed by the International Society for Electroencephalography, and it is widely utilized in EEG studies [100,101,102]. Most researchers adhere to the 10–20 international standard system for electrode placement during data acquisition, as illustrated in Figure 2. Table 4 summarizes the electrodes placed in different regions of the brain according to the 10–20 international standard system. The various brain regions utilized for EEG acquisition are depicted in Figure 3 [103]. An increasing number of researchers are focusing on EEG signals from specific brain regions. Acharya et al. [64] and Ay et al. [66] analyze EEG recordings from four electrodes, located in the left and right hemispheres of the brain. During data collection, Wan et al. [72] select several representative brain regions in the prefrontal cortex (PFC), frontal cortex, and parietal cortex—regions known for their strong association with depression—for EEG collection. To investigate inter-hemispheric asymmetry in patients with MDD, Duan et al. [35] utilize 28 pairs of electrodes to conduct experiments across five brain regions. The EEG signals used in the studies conducted by Wu’s team [74] and Xu’s team [73] are primarily collected from the frontal regions of the brain.

#### Analysis

The frequency of EEG signals typically lies below 100 Hz. According to Nyquist’s theorem, when the sampling frequency is more than twice the highest frequency of the signal, the sampled signal can retain the complete information of the raw signal. An examination of the depression diagnostic studies listed in Table 1 reveals that nearly all of them maintain a sampling rate exceeding 200 Hz. However, the indiscriminate use of a high sampling rate to garner more information may not be advisable. Processing data at high sampling rates demands increased computational resources, thereby substantially extending training times, particularly for deep learning models. Increased data volumes significantly raise storage needs and preprocessing times, potentially causing bottlenecks in both feature extraction and training phases. Additionally, higher-dimensional data from increased channel counts may increase the risk of model overfitting. Given that multi-channel data often contain substantial redundant information, models are likely to capture noise and irrelevant features rather than the intended signal patterns. Handling high-dimensional data often requires more complex model architectures, thereby increasing both the complexity of model training and the difficulty of tuning. In practice, researchers must balance sampling rates, channel counts, and model complexity to optimize the performance within the constraints of the available resources.

### 3.3. Preprocessing

**Figure 4:**
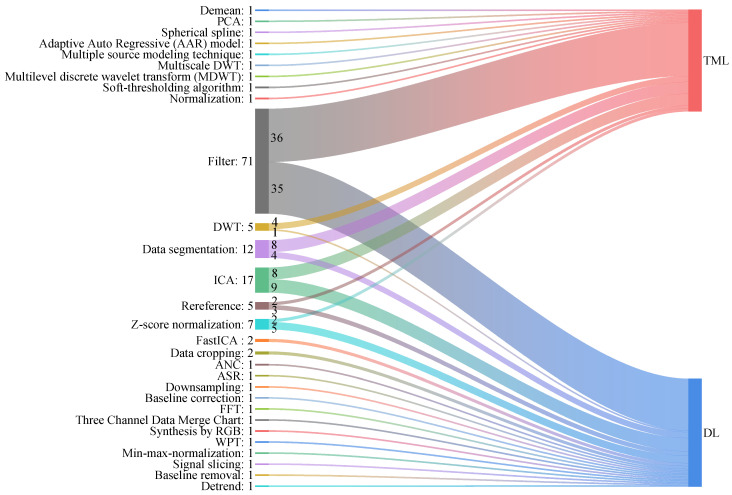
Frequency of different preprocessing methods used in studies of depression diagnosis.

Noise and artifacts must be filtered from EEG data prior to analysis [104]. EEG data inherently exhibit instability, weakness, and high susceptibility to external interference. Errors in experimental setups, environmental noise, and artifacts from other biological signals adversely impact the overall signal. Preprocessing constitutes a key component of EEG signal processing, significantly enhancing the signal-to-noise ratio and establishing a reliable foundation for further analyses and interpretation [105]. This article reviews a range of preprocessing methods employed in the included studies, including filtering, independent component analysis (ICA), data segmentation, Z-score and min–max normalization, principal component analysis (PCA), re-referencing, soft-thresholding algorithms, sphere spline, FastICA, adaptive autoregression (AAR) models, adaptive noise cancellation (ANC), pseudo-subspace reconstruction (PSR), discrete and multilevel discrete wavelet transforms (DWT and MDWT), fast Fourier transform (FFT), among others. Table 5 summarizes the preprocessing methods used in studies that employ both TML and DL approaches. The left column enumerates the various preprocessing methods, while the right column provides details of the studies that implemented these methods. Figure 4 graphically presents the frequency distributions of various preprocessing methods, with the numbers after the colon (“:”) denoting the usage frequency of each method. Furthermore, the thickness of each ribbon strip corresponds proportionally to its frequency.

As indicated in Table 5 and Figure 4, filtering is the most commonly employed preprocessing method. In all included studies on depression diagnosis, filtering was employed 71 times: 36 times in studies employing traditional machine learning and 35 times in those utilizing deep learning approaches. Various filter types employ specific window sizes and cutoff frequencies. Considering that the power line frequency in China is 50 Hz, researchers frequently use 50 Hz notch filters to reduce power line interference in recorded EEG signals. Bandpass, high-pass, and low-pass, each characterized by varying cutoff frequencies, are frequently employed as well. Kalman filters, adaptive filters, smoothing filters, and Hanning filters remain in the exploratory phase of application in this field. ICA is also a frequently employed method, utilized eight times in studies based on TML and nine times in those based on DL approaches. In contrast, FastICA was employed only twice in studies using deep learning approaches, as shown in Figure 4. This usage demonstrates FastICA’s faster convergence compared to traditional ICA and its suitability for deep learning tasks. ICA and FastICA are widely employed to isolate and remove disturbances attributable to eye and muscle movements [106,107,108,109]. Decomposing the EEG signal into its constituent components allows for the isolation and removal of artifacts by identifying and excluding these components [110]. A notable drawback of this artifact removal technique is its potential to alter the inherent signal dynamics. Research indicates that minimal preprocessing of physiological signals retains more of their original information [111]. To minimize signal alteration, some researchers engage EEG specialists who visually identify artifact-free segments for subsequent analysis [42].

Typically, the acquisition of EEG data requires special equipment and professional operation. Consequently, EEG data samples from depressed patients are often limited, especially in medical research, where acquiring large volumes of labeled data for depression diagnosis is challenging. Additionally, EEG data are characterized by nonlinearity, time variability, and sensitivity to noise, and must be processed prior to training deep learning models to avoid overfitting. Given the high temporal resolution of EEG signals, many researchers have employed data augmentation methods to improve the utility of limited data [112,113,114]. As illustrated in Figure 4, the method has been utilized in both TML and DL research contexts. Data segmentation, a technique frequently used in data augmentation, involves dividing a long EEG signal into multiple short time windows, thereby significantly increasing the amount of data available [115,116]. Segmentation improves the model’s focus on local features of the time series, thereby enhancing its performance. Researchers must consider the length of the segmentation window; a window that is too short risks losing global information, while one that is too long may not significantly increase the sample size. Some researchers have segmented EEG into specific time intervals using data cropping [81,117], a technique that enables researchers to concentrate on the electrical activity of the brain during certain key time windows. However, it is essential that data cropping ensures the selected time segments are sufficiently informative; otherwise, non-informative or noisy segments may be inadvertently included.

Z-score normalization is widely used to standardize EEG data across various channels or time periods to a consistent scale, thus eliminating discrepancies arising from varying amplitudes or baseline deviations [118,119]. Normalization improves the visibility of abnormal values within EEG signals, thereby facilitating the identification of atypical brain activity features. However, this method exhibits high sensitivity to outliers, such as noise from motion artifacts or electrode detachment. These outliers can substantially affect the calculation of means and standard deviations, consequently impacting the standardized results. Additionally, several studies have utilized specialized software and libraries to mitigate various types of noise, including Brain Electric Source Analysis (BESA), EEGLAB, Curry 7, Open MEEG toolbox, Brainstorm, ICLabel and MARA plugins, and MNE-Python.

#### Analysis

The preprocessing methods outlined above can be divided into two primary categories: automatic and semi-automatic or manual. In automatic preprocessing methods, tools such as EEGLAB and BESA rapidly process large volumes of EEG data, minimizing human intervention with techniques such as filtering, ICA, and normalization. This approach is especially well-suited for large-scale data analysis. Automated methods ensure consistency in preprocessing steps. For example, the use of uniform filters and standardization algorithms produces reproducible results across experiments, thereby enhancing the study’s reliability. FastICA and ANC effectively extract and remove artifacts and enhance signal-to-noise ratios, thereby improving signal quality. Typically, these methods can rapidly identify and eliminate artifacts caused by eye movements or other biological signals. However, automatic preprocessing can alter the intrinsic dynamics of the signal, especially when techniques such as ICA are employed [106]. Incorrect component identification may lead to the loss of crucial signal features. Among semi-automatic or manual preprocessing methods, the manual approach allows researchers to tailor the preprocessing strategy to specific experimental requirements. Expert visual inspection ensures the accuracy and integrity of the signal and minimizes potential distortions. Expert intervention facilitates more efficient identification and handling of outliers, thereby preventing the omission of important signals that may be overlooked by automated processing. This approach generally preserves more information and minimizes alterations to the signal. Although semi-automatic or manual methods can enhance data quality, insufficient sample sizes may lead to both the training and test sets containing the same individuals, thus impairing the model’s generalization capabilities. In conclusion, automatic preprocessing methods provide significant advantages in terms of efficiency and consistency, making them ideal for large-scale data analyses. However, they may also alter the signal characteristics. Conversely, semi-automatic or manual methods offer enhanced flexibility and improved control over signal quality, thereby allowing for the retention of more useful information. 

Although there are many similarities in EEG signal preprocessing between TML and DL approaches, notable differences in method selection and data processing strategies remain. In terms of method selection, deep learning predominantly employs sophisticated, automated techniques like ASR and ANC to effectively manage high-dimensional and complex data [120]. In contrast, traditional machine learning methods primarily employ classical signal processing techniques like PCA and Kalman filters, which concentrate on the statistical properties of signals and linear transformations. In terms of data processing methods, the transformation of EEG signals into images for convolutional neural network (CNN) analysis represents a novel approach, which is seldom employed in TML. This method underscores the capabilities of DL to effectively manage unstructured data, such as images and videos. In contrast, traditional machine learning prioritizes specific filtering techniques and models, such as Kalman filters and adaptive prediction filters, which are tailored for precise signal modeling and prediction. These differences highlight the unique needs and strengths of each approach in addressing data complexity and ensuring adaptability.

In summary, deep learning methods increasingly depend on automation and advanced techniques during preprocessing to enhance data quality and improve model performance. Traditional machine learning methods, known for their conciseness and efficiency, are particularly well-suited for environments with limited computing resources. These methods concentrate on fundamental signal characteristics and employ straightforward transformations during the preprocessing phase.

### 3.4. Feature Extraction

#### 3.4.1. Studies Based on TML Methods

Although EEG signals encompass a broad spectrum of information, the specific aspects related to depression remain unclear. Therefore, the signal must be optimized through the extraction of features that accurately represent the relevant information [121,122]. Feature extraction entails generating features by discerning hidden patterns within the input signal. In the reviewed literature, a range of methods have been employed to extract and select diverse types of features for diagnosing depression. Table 6 summarizes the various categories of features extracted from studies that employ traditional machine learning methods. As shown in Table 6, the investigated features are classified into five categories: time-domain, frequency-domain, nonlinear, connectivity, and generated features. Various methods have been utilized to extract and select diverse features for the diagnosis of depression, including FFT, DWT, MDWT, Neighborhood Component Analysis (NCA), Fourier–Bessel Series Expansion (FBSE), histogram-based feature generation, parametric modeling with autoregressive models, and detrended fluctuation analysis (DFA), among others.

Several researchers have introduced methods for automatic feature extraction. Several researchers have developed a graph where the nodes represent subjects within the dataset, employing the Node2vec algorithm to compute feature representations as node embeddings [44,45]. These node embeddings serve as valuable features readily applicable to classification algorithms. Aydemir’s team [54] pioneers the development of a molecular shape-based feature generator, termed the “melamine pattern”. In the approach, the melamine pattern is innovatively integrated with the DWT to generate texture features, also known as spatial domain features, significantly enhancing the classification accuracy. Tasci et al. [62] decompose the original signal using the Daubechies Four (DB4) parent wavelet function. They introduce an innovative Twin Pascal Triangle Lattice Pattern (TPTLP) approach to extract texture features from EEG signals. 

#### 3.4.2. Studies Based on DL Methods

TML methods depend on the manual design and selection of features, requiring the expertise and experience of domain specialists. Numerous researchers have attempted to remove this limitation by applying DL models to the field. During data processing, features are progressively extracted and integrated across multiple layers of the model, ultimately leading to a final decision or prediction based on these automatically extracted features [105]. However, deep learning models are often described as “black-box“, making their internal feature representations difficult to interpret. Furthermore, raw EEG signals may include information extraneous to depression, potentially impairing model performance if used directly as input. As a result, some researchers strive to integrate manually extracted features into deep learning models. In EEG signal processing, brain waves are typically categorized into four primary frequency bands: δ, θ, α, and β [123,124,125]. Each frequency band is associated with specific brain states and activities. Several researchers have focused their research on these frequency bands to enhance model performance through the extraction of structural features and power spectral density. Inspired by advances in deep learning for computer vision, several researchers have transformed EEG into image form, capitalizing on the strengths of DL in image processing to enhance EEG signal analysis. However, this approach could result in potential information loss, heightened processing complexity, and an increased risk of overfitting. Furthermore, given that the brain functional connectivity analysis reveals the interactions and synergistic activities among various brain regions [126,127,128], several researchers have combined brain functional connectivity matrices with feature fusion techniques. This approach increases the comprehensiveness of raw EEG signal information and provides substantial noise immunity. Furthermore, several researchers have utilized a range of techniques for EEG signal analysis, including Partial Directed Coherence (PDC), and the integration of temporal and spatial features, as well as both distance-based and non-distance-based projection methods. Table 7 summarizes the diverse feature extraction methods employed in combination with deep learning techniques.

#### 3.4.3. Analysis

An analysis of Table 6 shows that the features extracted in studies employing traditional machine learning methods are primarily time-domain features, with skewness and kurtosis being the most frequently utilized, followed by the mean. Among the nonlinear features, entropy calculation is a widely used technique, with approximate entropy and Shannon entropy also being frequently employed by researchers. Regarding the use of entropy, the EEG diagnosis of depression using DL methods has not yet been explored. Conversely, band power is the most frequently utilized feature in the frequency domain. Both approaches emphasize the critical role of functional connectivity in elucidating the brain’s functional networks. The application of molecular structure maps and lattices in EEG-based depression diagnosis represents an exceptionally innovative approach, providing significant potential for future exploration. As detailed in Table 7, studies employing deep learning methods frequently combine diverse feature extraction techniques with various deep learning models, including CNN, DAN, LSTM, GNN, and MLP. 

Features extracted through traditional machine learning methods are typically low-level and directly derived from the original signal. Deep learning methods can extract high-level features across multiple layers, producing more abstract and complex representations that significantly improve model performance. Traditional machine learning features are crafted for straightforward interpretation, while deep learning models exhibit greater complexity and reduced interpretability. In their experiments, researchers often employ visualization techniques, including activation maps and feature maps, to aid interpretation. In summary, while deep learning offers robust capabilities for automatic feature extraction and learning in EEG signal processing, its complexity and the significant computational resources it demands must also be considered.

### 3.5. Feature Selection

Feature selection reduces feature dimension to remove redundant or irrelevant features. The extracted features may be redundant or irrelevant. Utilizing all of these as inputs to the classifier could potentially decrease its accuracy. To improve classifier accuracy, key features must be selectively identified and utilized in the analysis [129]. Table 8 presents a summary of the feature selection methods employed in EEG-based depression diagnosis studies. According to Table 8, Neighborhood Component Analysis (NCA) emerges as the most frequently employed method. The primary objective of Neighborhood Component Analysis (NCA) is to enhance classification performance through the development of a feature transformation matrix. This matrix aims to bring samples from the same category closer together, and to separate samples from different categories within the transformed feature space. However, NCA exhibits significant computational complexity, demonstrates sensitivity to noise and outliers, and is optimally suited for analyzing linear feature relationships. Additionally, methods such as the genetic algorithm (GA), ReliefF, Node2vec, and others are commonly utilized.

Certain DL models, including CNN, LSTM, and Recurrent Neural Networks (RNNs), automatically filter and prioritize input features through their inherent structure and training process. This method effectively highlights useful features while minimizing the impact of redundant or irrelevant ones. Additionally, certain deep learning models employ regularization techniques, such as L1 and L2 regularization, Dropout, and Batch Normalization, to mitigate overfitting and indirectly facilitate feature selection. Visualization tools, such as Grad-CAM and t-SNE, facilitate researchers’ interpretation of model features and regions of interest, thereby enhancing feature selection and analysis.

In summary, deep learning-based methods eliminate the necessity for manual feature selection and excel at processing large-scale, high-dimensional, and complex data. However, the feature selection process in deep learning models is frequently viewed as a black-box, complicating efforts to interpret the decision-making logic within the model. Feature selection continues to be a critical area of research, substantially affecting classification performance and playing a crucial role in subsequent data analyses.

### 3.6. Classification Methods

#### 3.6.1. Studies Based on TML Methods

Table 9 summarizes the performance of each algorithm in studies of depression diagnosis that use traditional machine learning methods. From left to right, the table lists the algorithm used, the validation strategy (which details the proportion of the dataset allocated to training, testing, and validation), the top-performing classifier, the accuracy given as a percentage, and the associated study. The choice of classifiers significantly influences the performance of the models, with various studies employing distinct classification methods. Currently, a diverse array of traditional machine learning algorithms is systematically applied to the diagnosis of depression and categorized into seven primary groups:

1. Support Vector Machine-Based Classifiers: this category includes the Support Vector Machine (SVM) with various kernels and Least Squares-SVM (LS-SVM).
2. Tree-Based Structures: features methods such as Decision Tree (DT), Best-First Tree (BF-Tree), and Coarse Tree.
3. K-nearest neighbor (KNN) and variants: encompasses KNN-GA and E-KNN.
4. Ensemble Learning Methods: comprises Bagging, Extreme Gradient Boosting (XGBoost), GentleBoost (GB), RusBoost (RB), Random Forest (RF), and Adaptive Boosting (AdaBoost).
5. Probabilistic models: includes well-known methods such as Naïve Bayesian (NB) and Logistic Regression (LR).
6. Discriminant Analysis-Based Classifiers: features Linear Discriminant Analysis (LDA).
7. Other methods: includes approaches such as K-means Singular Value Decomposition (K-SVD) and Linear Regression.

**Figure 5:**
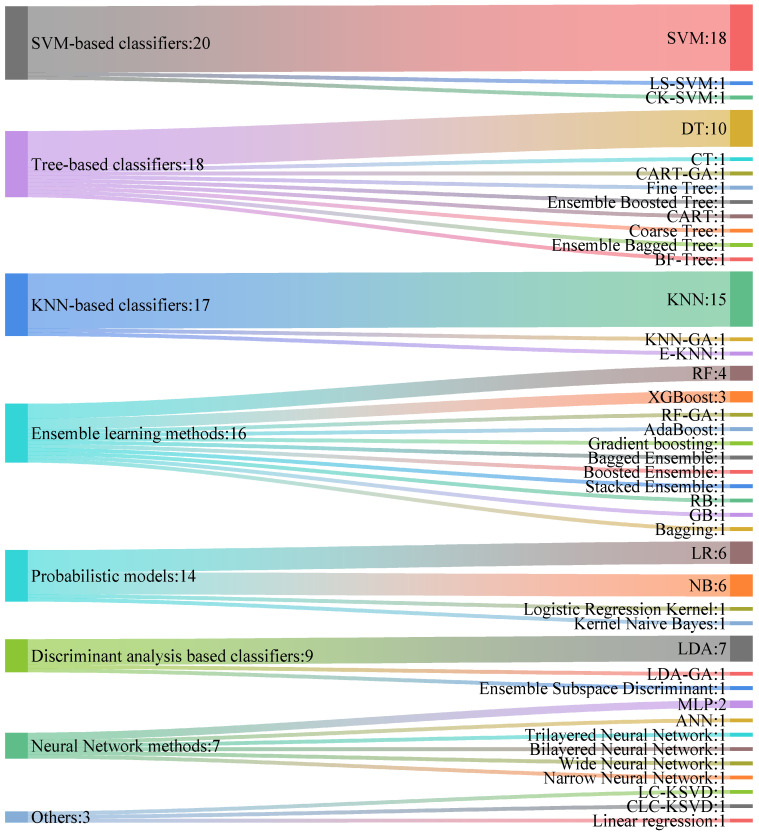
Frequency distribution of TML-based algorithms for depression diagnosis.

Figure 5 illustrates the frequency distribution of depression diagnosis algorithms that utilize traditional machine learning, with the numbers following the colon (“:”) indicating the frequency of usage for each method.

##### Analysis

This review mainly analyzes the classification accuracy of various studies. Classification accuracy represents the percentage of all participants who were correctly classified as either depressed or in the control group. As illustrated in Figure 5, KNN and SVM were the classifiers most frequently used in the included studies on depression diagnosis, with KNN appearing 15 times and SVM 18 times. As shown in Table 9, KNN and SVM emerged as the top-performing classifiers in several studies. On non-public datasets, the KNN classification demonstrated a strong performance, with accuracies ranging from 76.83% to 98.43%. On public datasets, the KNN classification yielded superior results, surpassing other classifiers such as BF-Tree and K-SVD in studies using the MODMA dataset [61] and the Mumtaz team’s dataset [39,50,51], achieving peak classification accuracies of 100% and 99.11%, respectively. This reflects the model’s exceptionally high performance under specific conditions; however, this does not rule out the possibility of sample leakage, which could artificially inflate the classification accuracy. SVM also exhibits an outstanding performance, with accuracy rates ranging from 83.67% to 98%. Probabilistic models like LR, as well as LDA, have also been explored somewhat in this area; however, none have emerged as the top-performing classifiers in any of the studies reviewed. Furthermore, ensemble methods have exhibited high accuracies, underscoring the advantages of integrated learning approaches for complex tasks, achieving a maximum accuracy rate of 99.11%. As shown in Figure 5 and Table 9, among tree-based classifiers, Decision Tree is not only the most frequently used algorithm, but it also ranks as the highest-performing classifier in several studies, with accuracies reaching up to 95%.

In summary, the accuracies reported in the referenced studies varied from 76.83% to 100%. For traditional machine learning methods, the classification accuracy depends more on the selected features than on the choice of classifier [130]. Many studies focus on examining the impact of diverse feature extraction methods on model performance. Some studies aim to reduce system complexity by minimizing the number of channels used, while still preserving accuracy. Although many studies report respectable classification accuracies, they often involve a small number of participants, which may limit the model’s generalizability and its applicability in clinical application.

#### 3.6.2. Studies Based on DL Methods

Table 10 summarizes the performance of various algorithms used in DL-based depression diagnosis studies. From left to right, the table lists the algorithms employed, the validation strategy (which details the percentage of the dataset allocated to training, testing, and validation), the top-performing classifier, the accuracy presented as a percentage, and the associated study. Deep learning algorithms for diagnosing depression in the reviewed studies are categorized into four primary groups:

1. CNN and variants, including CNN, 2D-CNN, CNN-LSTM, Lightweight-CNN, AchCNN, and EEGNet;
2. Graph Convolutional Network (GCN) and variants, such as GCN, MGGCN, GICN, and AMGCN;
3. Recurrent Neural Network (RNN) and variants, including RNN, 4D-CRNN, GRU, and GC-GRU;
4. Inception-based models and variants, notably Inception, InceptionTime, InceptionNet, and Tsception.

**Figure 6:**
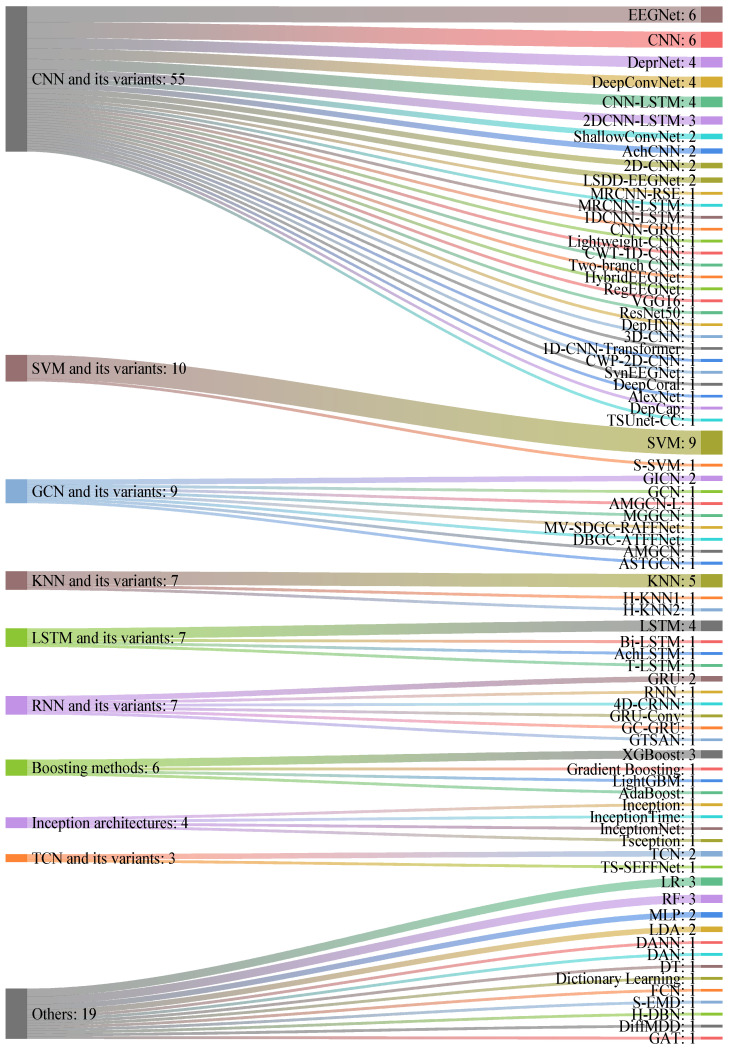
Frequency distribution of deep learning-based algorithms for depression diagnosis.

Among these, numerous hybrid models are identified, including DeprNet [65], AchLSTM [66], AchCNN [64], T-LSTM [131], H-KNN1 [38], H-KNN2 [132], S-EMD [133], S-SVM [134], H-DBN [135], EEGNet [136], DeepConvNet [137], ShallowConvNet [137], LSDD-EEGNet [70], HybridEEGNet [72], SynEEGNet [72], RegEEGNet [72], MRCNN-RSE [73], MRCNN-LSTM [73], DeepCoral [138], VGG16 [139], AlexNet [139], ResNet50 [139], MV-SDGC-RAFFNet [84], TS-SEFFNet [140], 4D-CRNN [141], DBGC-ATFFNet [142], ASTGCN [143,144], DepHNN [145], TSUnet-CC [87], DiffMDD [88], 1D-CNN-Transformer [66,145], CWT-1D-CNN [146], CWP-2D-CNN [146], GRU-Conv [147], and GC-GRU [148]. Figure 6 illustrates the frequency distribution of DL-based algorithms used in diagnosing depression, with the numbers following the colon (“:”) indicating the usage frequency of each method.

##### Analysis

As shown in Figure 6, CNN and its variants constitute the most commonly employed deep learning method in the research. An analysis of Table 10 shows that most studies utilizing CNN and its variants (e.g., 2D-CNN, CNN-LSTM) exhibit an excellent performance. In studies using publicly available datasets from Mumtaz’s team [39,50,51,89] and Cavanagh’s team [60], CNN variants (2D-CNN and Lightweight-CNN) surpassed other classifiers in attaining the highest classification accuracy. Among these, the 2D-CNN is particularly notable, with Khan’s team [80] achieving a 100% classification accuracy in the study, which highlights its exceptional capability in EEG signal processing. The accuracy of Lightweight-CNN has reached 99.87% and reduced computational resource consumption while preserving high accuracy, rendering it adaptable for a wide range of applications. The CNN-LSTM model merges the feature extraction capabilities of CNN with the time series processing advantages of LSTM, attaining an accuracy of up to 99.9%. LSTM variants excel in processing EEG signals but prove relatively ineffective when used independently. Hybrid models, including MV-SDGC-RAFFNet and TSUnet-CC, which integrate the strengths of multiple models, achieve accuracies of 99.19% and 99.22%, respectively, highlighting their potential for future hybrid model development. The application of GCN and its variants in EEG signal processing has shown excellent accuracy (see Table 10). In studies utilizing the public dataset MODMA [61], MGGCN delivered the highest performance, achieving an accuracy of 99.69% [76]. The application of GCN and its variants in EEG signal processing merits additional exploration. MLP delivered the highest performance in the study utilizing the public dataset EDRA.

In summary, deep learning methods consistently outperform traditional machine learning techniques in classification accuracy, demonstrating substantial potential for EEG-based automatic depression diagnosis. However, the black-box-like properties of DL models complicate the interpretation of their decision-making processes, potentially reducing their trustworthiness in clinical applications. Studies utilizing various publicly available datasets have yielded diverse optimal classifiers, indicating that the existing findings lack sufficient generalizability. Gathering more heterogeneous data and performing additional experimental comparisons will help address this issue.

### 3.7. Validation Methods

**Figure 7:**
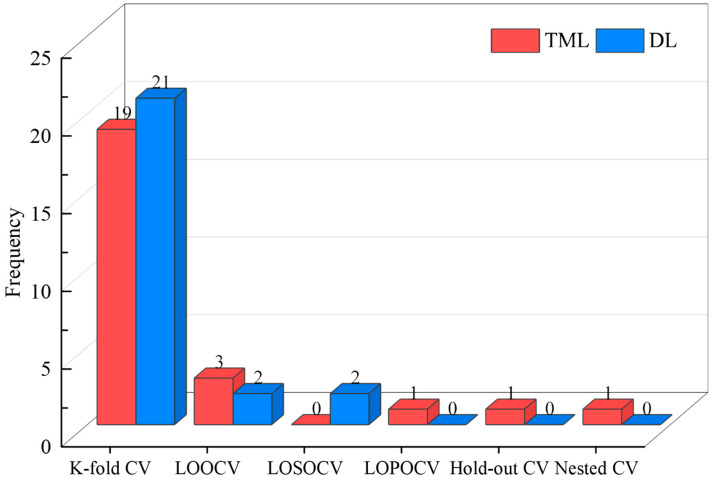
Frequency distribution of different validation methods in depression diagnostic studies.

To detect overfitting and ensure model generalization, it is essential to evaluate both traditional and deep learning models using suitable validation techniques. Figure 7 depicts the frequencies of various validation methods used in depression diagnostic studies, with the numbers above the bars showing the frequency of each method. As depicted in Figure 7, among the included depression diagnostic studies, the n-fold cross-validation (CV) is the most frequently employed method by researchers, succeeded by the LOOCV. The n-fold CV [149,150,151], typically configured for 5 or 10 folds, is the approach most commonly employed by researchers (refer to Table 9 and Table 10). This method is the most commonly utilized validation technique, and numerous models in this study exhibit a strong performance when assessed using this approach. Specifically, across n iterations, each sample is included n − 1 times in the training set and once in the validation set. This method effectively utilizes the entire dataset and mitigates the issue of small sample sizes prevalent in many studies. However, n-fold CV presents certain drawbacks, including significant computational costs attributed to multiple training and evaluation cycles. Additionally, if the dataset size is not divisible by n, this can lead to varying fold sizes, potentially impacting the accuracy of the evaluation results. To address this issue, several studies have chosen to partition the dataset on a per-subject basis. However, this validation method entails the risk that certain sample types may exclusively appear in either the training or the testing set, especially in cases of imbalanced datasets. Wan’s team [40] implemented a no-putback sampling strategy in their study, ensuring consistent sample sizes for both the depressed and normal control groups, thereby addressing the issue of model performance degradation due to sample imbalance.

To optimize information utilization from each sample, several studies have implemented LOOCV [152] and its variants, including LOSOCV and LOPOCV [47,58,62]. LOPOCV and LOSOCV are well-suited for datasets with substantial individual differences, while LOOCV is more appropriate for datasets with small sample sizes. Additionally, one study [54] implements the hold-out validation strategy [153], which entails randomly partitioning the dataset into two mutually exclusive subsets: one designated for training and the other for testing. If the test set is too small, the evaluation results might be unstable and lack accuracy; conversely, an overly large test set can undermine the realism of model training. Furthermore, hold-out validation can introduce bias from the data partitioning process, particularly when significant differences exist between the sample proportions in the training and testing sets. Maintaining data independence is crucial when employing these methods to preserve the accuracy and reliability of model assessments. However, owing to limited sample sizes in many studies, the test and training sets frequently lack independence, potentially resulting in data leakage. Wu’s team [41] mitigates this issue by implementing 5-fold CV and validating the model with an independent test set, thus minimizing the risk of data leakage. A research team [63] has used the nested CV strategy [154], effectively reducing data leakage by separating parameter selection from performance evaluation, thus providing a more robust assessment of the model’s generalization capability. However, this strategy incorporates two layers of cross-validation, which may complicate its implementation.

When selecting a validation strategy, researchers should base their decisions on factors including dataset size, computational resources, model complexity, and evaluation requirements. For large-scale datasets constrained by limited computational resources, hold-out CV provides a rapid and straightforward method. For medium-sized datasets, k-fold CV, such as 5-fold or 10-fold, ensures a more stable evaluation. When dealing with small datasets and ample computational resources, LOOCV optimizes data utilization. For complex models and hyperparameter tuning, nested CV is the preferred method, providing low-bias model evaluations and optimized generalization error estimations. In scenarios involving unbalanced datasets, stratified k-fold or stratified nested CVs may be utilized to ensure consistent sample proportions across categories. Future research should emphasize the utilization of large datasets and independent samples wherever feasible. 

## 4. Discussion

Researchers in existing studies commonly utilize two types of methods: TML and DL. Numerous studies report substantial accuracy in diagnosing depression using rsEEG. Given the distinctions between the two research methods, we opted to investigate the commonalities among these studies.

To begin with, accurately identifying depression is the first challenge that researchers must address. Episodes of MDD often coexist with other disorders, such as bipolar disorder and anxiety disorders [155]. The clinical manifestations of major depression and bipolar disorder are challenging to distinguish [156,157,158]; moreover, most individuals with bipolar disorder also experience phases of major depression [159,160]. Recent research has revealed that resting-state connectivity biomarkers are capable of defining neurophysiological subtypes of depression [161]. Future research may explore solutions to this issue through EEG-based, data-driven approaches [162].

Various studies may utilize differing diagnostic tools and criteria, leading to heterogeneity in EEG parameters across subjects. Consequently, identifying reliable biomarkers for MDD based on EEG signals is crucial [163,164,165]. Several researchers have investigated the feasibility of employing wavelet coherence between regions of DMN as a biomarker [80]. Moreover, research has shown that both effective connectivity and functional connectivity among various brain regions are essential for accurately distinguishing between depressed patients and healthy participants [79,86,166,167].

Currently, numerous studies employing TML and DL methods with EEG data face the pervasive issue of insufficient sample sizes. Among the studies included in the comparative analysis, only four featured a sample size exceeding 200 cases. Most studies exhibited small to moderate sample sizes and relied on limited sample sources. A large sample size is typically not derived from a single location; rather, data are gathered from multiple sources, including hospitals, clinics, neuroscience research institutes, clinical trials, research programs, and universities. Furthermore, the range of subjects’ occupations could be broadened to include IT workers, doctors, video bloggers, business managers, laborers, and sales personnel. Recently, advancements in portable EEG recording devices have streamlined the collection of EEG data [168,169]. For instance, wireless EEG caps such as the Epoch, ENOBIO Neuroelectrics, and iMotions not only offer portability and comfort, but they also facilitate real-time data transmission. The enhancement of public databases and the development of standardized datasets have offered essential support for research in depression diagnostics. Mumtaz’s team [39,50,51,89], Cavanagh’s team [60], Cai’s team [61], Yang’s team [90], and researchers including Wang et al. [98], and Kang et al. [77] have publicly shared their datasets or code, thereby facilitating knowledge sharing. Many researchers have expanded upon these contributions. We encourage researchers in this field to share their data and code. Doing so not only promotes scientific transparency and reproducibility, enhances research credibility, and facilitates the verification and reproduction of results by others, but it also fosters cooperation and communication, thereby advancing technological innovation in this area [170].

The limited availability of shared datasets in this field can be partly attributed to the complexity of EEG signal acquisition. In clinical practice, EEG signals are typically acquired using traditional wet electrodes [171]. However, the requirement to apply a conductive medium, such as conductive paste, between the electrodes and the scalp complicates the procedure and creates discomfort for the subject. Dry and semi-dry electrodes offer a more efficient alternative for EEG signal acquisition, especially in situations where professionals are unavailable [172]. Currently, newly developed semi-dry gel electrodes offer greater comfort compared to wet electrodes, and they address the issue of rigid dry electrodes not adhering stably to the skin [173,174]. This advancement simplifies the EEG collection process and enhances the multifunctionality of the technology across a range of scenarios. The widespread adoption of gel electrodes could significantly enhance EEG signal acquisition.

KNN and SVM are the classifiers most frequently utilized in studies employing traditional machine learning methods. Meanwhile, studies that utilize deep learning methods frequently employ various types of CNNs and their variants. These models have demonstrated a robust performance in diagnosing depression, achieving exceptionally high accuracy rates. To enhance the generalization capabilities of the models, researchers frequently employ n-fold CV and LOOCV methods for evaluation. However, most studies were not tested with independent samples, and some lacked the explicit reporting of validation information, casting doubt on the reliability of the model results. Future studies should ensure the complete independence of training and test sets and should transparently report their distributions to affirm the reliability and clinical applicability of the model results. Generalization ability denotes a model’s capacity to predict and process new data beyond the training dataset. Consequently, external validation is essential. Among the included studies on depression diagnosis, none were found to have developed a model on one dataset (its own dataset) and validated it on another (a public dataset). Implementing such a process would offer a robust test of the model’s generalizability. Only a few existing studies have considered using multiple datasets, and they mostly rely on commonly used public datasets (e.g., MODMA [61]), potentially affecting model robustness. The subsequent phase entails the development of models on proprietary datasets, followed by their validation on datasets that are publicly accessible. Furthermore, the model must be interpretable [175]. With highly interpretable models, researchers can determine which EEG features, including activity in specific frequency bands, most significantly influence diagnostic outcomes. This understanding facilitates the more precise identification of depression biomarkers, offering essential insights for advancing neuroscience research. Enhancing model interpretability presents a substantial challenge for deep learning-based approaches. Researchers must meticulously select algorithms based on their intended applications. The development of visualization tools and interpretative algorithms can aid clinicians in comprehending and applying the results derived from studies employing CNN architectures. Moving forward, it is imperative to intensify comparative studies among various models to identify optimal solutions that improve current clinical practices and provide more effective treatment strategies for patients with depression.

Presently, methods that diagnose depression via a single EEG signal still encounter significant limitations regarding accuracy and clinical applicability. Multimodal approaches to EEG are generally more effective than unimodal methods, as computer vision techniques can extract features more efficiently by leveraging multiple models, rather than relying on a single model [132,176,177,178,179]. Future research should investigate the integration of multimodal data, incorporating a variety of sources including speech, facial expressions, text, images, eye tracking, RNA-Seq, functional magnetic resonance imaging (fMRI), and other modalities to enhance the diagnostic accuracy and generalizability [18,164,180,181,182,183,184,185,186,187,188,189,190,191].

Classifying the subjects as those with MDD or HC represents only the preliminary step in depression research employing EEG signals. The development of predictive models for depression treatment outcomes and relapse is crucial for preventing ineffective treatment strategies [192,193,194,195,196,197,198]. Furthermore, the use of IoT technology in developing personalized medical models, which take into account individual differences and clinical characteristics, offers considerable potential for providing precise diagnoses and tailored treatment plans for patients with depression [199,200].

As this research advances, computational intelligence-based diagnostic systems for depression are anticipated to exert a growing influence on clinical applications, offering more reliable and intelligent diagnostic and treatment solutions. Simultaneously, these technological advances are also expected to yield valuable insights into the diagnosis and research of other mental disorders.

## 5. Conclusions

The diagnosis of depression remains a significant and challenging issue in contemporary medical research. Diagnostic methods traditionally depend on clinical assessments and self-reports; however, these approaches often face challenges stemming from subjectivity and clinical bias. In recent years, the advent of computational psychiatry has catalyzed a growing number of studies that integrate electroencephalography with artificial intelligence, substantially enhancing the diagnosis of depression. In this review, we conduct a comparative analysis of 49 studies that utilize rsEEG in conjunction with both TML and DL techniques to diagnose depression.

EEG is capable of detecting specific brain physiological changes related to depression, offering a crucial objective foundation for depression diagnosis. TML methods are extensively employed in the diagnosis of depression, notably demonstrating significant advancements in feature extraction. Although feature extraction is complex and time-intensive, traditional machine learning offers superior interpretability in analyzing diagnostic results compared to deep learning methods. Commonly used traditional machine learning algorithms, including KNN and SVM, have demonstrated an excellent classification performance in numerous studies. Recently, research on the application of DL methods for diagnosing depression has expanded significantly. Deep learning demonstrates significant potential in processing raw EEG signals due to its capability for automatic feature extraction. CNN and its variants represent the most commonly utilized deep learning architectures. By integrating manually extracted features with these models, researchers further enhance the accuracy of depression diagnosis. However, deep learning-based studies still face limitations due to the scarcity of large-scale EEG datasets, and the challenge of model interpretability persists. Furthermore, validating these models using independent datasets is crucial to ensure their generalization capabilities. Going forward, researchers will focus more on multimodal data fusion methods. Currently, the integration of artificial intelligence and EEG in depression research offers new opportunities for precision medicine and preventive strategies. We anticipate a growing number of individuals benefiting from this advancement.

## Figures and Tables

**Table 1:** Basic experimental setup for EEG data acquisition in diagnostic studies of depression.

Researches Based on TML Methods			
Sample Size (MDD + HC)	Frequency (Hz)	Electrodes	Study
92 + 121	250	3	[38]
34 + 40	250	19	[39]
23 + 12, 15 + 15	500, 512	2, 1	[40]
200 + 200	500	26	[41]
10 + 10	200, 400	18	[42]
92	-	19	[43]
23 + 33, 24 + 29, 15 + 18	250, 256, 512	3, 128, 19	[44]
23 + 33, 24 + 29, 15 + 18, 32 + 30	250, 250, 512, 256	3, 128, 19, 19	[45]
15 + 18	256	22	[46]
49 + 49	1000	64	[47]
19 + 13	128	14	[48]
40 + 40	1000	32	[49]
34 + 30	256	19	[52]
34 + 30	256	19	[53]
34 + 30	256	19	[54]
34 + 30	250	19	[55]
30 + 30	256	19	[56]
24 + 25	256	19	[57]
34 + 30	250	19	[58]
42 + 42	500	2	[59]
24 + 29	250	128	[62]
26 + 29	-	3	[63]
Research Based on DL Methods			
Sample Size (MDD + HC)	Frequency (Hz)	Electrodes	Study
34 + 30	256	19	[36]
15 + 15	256	4	[64]
15 + 18	256	19	[65]
15 + 15	256	4	[66]
24 + 24	250	128	[67]
34 + 30	256	19	[68]
40 + 40	1000	3	[69]
40 + 40	1000	3	[70]
30 + 28	256	19	[71]
23 + 12	500	6	[72]
41 + 34	250	6	[73]
16 + 16	-	56	[35]
26 + 29	250	3	[74]
24 + 29	125	64	[75]
23 + 25, 23 + 25, 40 + 67	250, 256, 500	128, 19, 66	[76]
34 + 30	256	19	[77]
34 + 30	256	19	[78]
30 + 30	256	19	[79]
30 + 30	256	19	[80]
17 + 17	-	128	[81]
26 + 24, 24 + 29	500, 250	62, 128	[82]
24 + 29, 26 + 24	250, 500	128, 62	[83]
24 + 29, 34 + 30	250, 256	128, 19	[84]
20 + 32, 24 + 29	500, 250	66, 128	[85]
27 + 28, 22 + 22	256, 256	19, 19	[86]
28 + 24	256	19	[87]
34 + 30, 23 + 19	256, 256	19, 19	[88]

**Table 2:** Publicly available datasets used in diagnostic studies of depression.

Public Datasets	Network Source	Study	
		TML	DL
Mumtaz et al. [39,50,51,89]	https://figshare.com/articles/dataset/EEG_Data_New/4244171, (accessed on 15 October 2024)	[52,53,54,55,56,57,58]	[36,67,68,71,74,76,77,78,79,80,82,83,84,85,86,87,88]
MODMA [61]	http://modma.lzu.edu.cn/data/index/, (accessed on 15 October 2024)	[44,45,62,63]	[67,74,76,82,83,84,85]
Cavanahg et al. [60]	https://openneuro.org/datasets/ds003478/versions/1.1.0, (accessed on 15 October 2024)	[59]	[76,86]
EDRA [82]	https://github.com/EllieYLJ/, (accessed on 15 October 2024)	[82]	[82,83]
PRED + CT	http://predict.cs.unm.edu/downloads.php, (accessed on 15 October 2024)	-	[85,88]

**Table 3:** Detailed information on non-public datasets used in diagnostic studies of depression.

Studies Based on TML Methods				
Male/Female	Age Mean or Age Range	Diagnostic Criteria for MDD	Source	Study
-	-	MMSE	-	[38]
MDD: 11/12 HC: 6/6, MDD: 9/6 HC: 11/4	MDD: 29.3 HC: 26.4, MDD: 34 HC: 33.6	DSM-IV HAM-D MINI	Beijing Anding Hospital, China	[40]
MDD: 58/142; HC: 58/142	MDD: 53.88 HC: 52.23	DSM-5 MINI	NTUH TVGH CGMHKL CGMHLnK	[41]
6/14	24–60	HAM-D EST-Q	-	[42]
-	-	HAM-D	Shenzhen Traditional Chinese Hospital	[43]
MDD: 11/4 HC: 16/2	MDD: 21.86 HC: 21	PHQ-9	-	[46]
0/98	MDD: 45.39 HC: 48.18	DSM-5 HAM-D	The Psychiatry Department of Inje University Ilsan Paik Hospital	[47]
16/16	MDD: 21.6 HC: 21.3	PHQ-9	Independent University, Bangladesh	[48]
MDD: 14/22 HC: 14/17	MDD: 22.7 HC: 25.2	MINI HAM-D	The Affiliated Brain Hospital of Guangzhou Medical University	[49]
Studies based on DL methods				
Male/Female	Age mean or age range	Diagnostic criteria for MDD	Source	Study
-	20–50	Clinical diagnosis	The Psychiatry Department, Medical College, Calicut, Kerala, India	[64]
-	-	PHQ-9	-	[65,66]
MDD: 15/25 HC: 17/23	MDD: 45.5 HC: 44.9	ICD HAM-D	The Second Affiliated Hospital of Jining Medical College	[69,70]
MDD: 11/12 HC: 6/6	MDD: 29.2 HC: 26.4	-	Beijing Anding Hospital, China	[72]
MDD: 10/31 HC: 11/23	MDD: 45.22 HC: 40.18	HAM-D	The designated hospital for psychosis and the local community	[98]
MDD: 7/9 HC: 7/9	MDD: 31.0 HC: 26.1	DSM-IV HAMD	Beijing Anding Hospital, China	[35]
MDD: 13/11 HC: 20/9	MDD: 30.88 HC: 31.45	PHQ-9 LES GAD SSRS PSQI	Second Hospital of Lanzhou University in Gansu, China, and Lanzhou city of Gansu Province using posters	[75]
MDD: 11/6 HC: 13/4	MDD: 33.35 HC: 30.29	MINI PHQ-9	Lanzhou University Second Hospital	[81]

MMSE: Mini Mental State Examination, NTUH: National Taiwan University Hospital, TVGH: Taipei Veterans General Hospital, CGMHKL: Chang Gung Memorial Hospital KeeLung, CGMHLnK: Chang Gung Memorial Hospital LinKou, LES: Life Event Scale, GAD: Generalized Anxiety Disorder Scale, SSRS: Social Support Research Scale, and PSQI: Pittsburgh Sleep Quality Index.

**Table 4:** Electrodes placed in different brain regions in the 10–20 international standard system.

Brain Region	Electrodes
Frontal	Fp1, Fp2, Fz, F3, F4, F7, F8
Temporal	T3, T4, T5, T6
Parietal	P3, P4, Pz
Occipital	O1, O2, Oz
Central	C3, C4, Cz
Reference points electrodes	A1, A2

**Table 5:** Preprocessing methods used in depression diagnostic studies.

Researches Based on TML Methods	Study
Finite Impulse Response (FIR) filter	[38,57,59]
DWT	[38,40,54,55]
Multiscale DWT, Kalman filter, Adaptive Predictor Filter (APF), Adaptive Auto Regressive (AAR) model	[38]
Multiple source modeling technique	[39]
Notch filter	[44,45,46,49,53,55,63]
Z-score normalization/Z-score standardization	[40,43]
Data segmentation	[40,42,48,52,53,56,58,63]
Adaptive filter, soft-thresholding algorithm	[40]
Bandpass filter	[41,42,47,49,55,56]
ICA	[41,46,49,52,53,56,57,59]
Low-pass filter	[44,45,46,52,53,57,58]
Normalization, Demean	[46]
PCA	[47]
IIR Butterworth filter, Spherical spline	[48]
Rereference	[49,56]
High-pass filter	[44,45,46,52,53,57,58,59]
Multilevel discrete wavelet transform (MDWT)	[62]
Smoothing filter	[53]
Research based on DL methods	Study
Bandpass filter	[36,68,71,73,75,76,78,79,80,85,86,87]
Notch filter	[36,64,65,66,67,68,69,70,71,78,79,80,86]
ICA	[35,36,65,73,77,78,83,85,87]
Z-score normalization	[64,71,72,76,78]
High-pass filter	[65,88]
Low-pass filter	[65,84,88]
Adaptive noise cancellation (ANC)	[67,75]
Artifact subspace reconstruction (ASR)	[68]
Wavelet threshold filter	[69,70]
Finite Impulse Response (FIR) filter	[35,70]
Rereference	[71,76,87]
Downsampling	[73]
Baseline correction	[73]
Data segmentation	[73,77,78,82]
FFT	[35]
Three Channel Data Merge Chart, Synthesis by RGB	[74]
DWT	[75]
FastICA	[76,81]
Wavelet packet transformation (WPT)	[76]
Min–max normalization	[77]
Hanning filter	[81]
Data cropping	[81,82]
Signal slicing	[82]
Baseline removal	[84]
Detrend	[84]

**Table 6:** Different classes of features extracted in studies based on traditional machine learning methods.

Time-domain features	
Feature extraction method	Study
Peak	[38]
Skewness	[38,40,48,52,58,59,63]
Kurtosis	[38,40,48,52,58,59]
Mean/average	[40,46,52,58,63]
Standard deviation (SD)	[40]
Maximum, minimum	[40,52,58]
Median	[40,46,58]
Variance	[38,48,58,59]
25th and 75th percentile	[40]
Mode	[46,58]
Mean cube, standard deviation, first difference, normalized first difference, second difference, normalized second difference, mobility, complexity, and Pearson’s coefficient of skewness	[46]
Range, first quartile, third quartile	[58]
Hjorth parameters (activity, mobility, and complexity)	[38,48,52,59]
Standard deviation	[58,63]
Relative median, root mean square	[59]
Maximum and minimum amplitude, peak-to-peak signal value, peak-to-peak time, mean of absolute values of first and second difference	[63]
Frequency-domain features	
Feature extraction method	Study
Relative centroid frequency, absolute centroid frequency relative power, and absolute power	[38]
Power spectrum, 8-level DWT, and the square summation of the detailed coefficients	[40]
Band power (BP)	[41,46,52,55,56,59]
Coherence	[41]
Alpha power variability, spectral asymmetry index	[42]
β/α ratio	[43]
Global relative powers, relative fronto-central powers, alpha asymmetry 1, alpha asymmetry 2	[46]
Power spectral density (PSD)	[47]
Power spectrum	[49]
Interhemispheric asymmetry, DWT	[52]
Wavelet packet decomposition	[55]
Relative band power	[42,59]
Mean frequency, median frequency, PSD, maximum-PSD, singular value	[59]
Energy	[63]
Nonlinear features	
Feature extraction method	Study
Kolmogorov entropy	[38,52]
Shannon entropy	[38,46,48,52,58,59,63]
Approximate entropy	[40,52,55,56,58]
Wavelet entropy	[40]
Higuchi’s Fractal Dimension (HFD)	[41,42]
C0-complexity	[38,40,52]
Katz’s Fractal Dimension (KFD)	[41]
Lempel–Ziv complexity (LZC)	[42,57]
Power spectral entropy	[38,40]
Detrended fluctuation analysis (DFA)	[42,52,56]
Log energy entropy	[48]
Higuchi, correlation dimension, Lyapunov exponent	[52]
Correlation dimension (CD)	[38,56]
Sample entropy	[55,56]
Dispersion entropy, zero crossing rate	[58]
Connectivity features	
Feature extraction method	Study
Synchronization likelihood (SL)	[39,52,53]
Node embeddings	[44,45]
Functional connectivity	[47,49]
Network indices	[47]
Feature generation	
Feature extraction method	Study
Melamine pattern and DWT-based multileveled feature generation	[54]
TPTLP-based feature extraction	[62]

**Table 7:** Different feature extraction methods combined with deep learning methods.

Feature Extraction Methods	Architectures	Study
Combining the structural features and connectivity features	CNN	[35]
Convert EEG signals into images	DAN	[74]
Fusion of temporal and spatial domain EEG features	3 layer LSTM + 4 layer CNN	[75]
Learning topological features of changes between functional brain regions and brain salience patterns on multi-granular functional neural networks using graph neural networks	GNN	[76]
Converting asymmetric features of EEG to matrix images	2D-CNN	[77]
Generating Spectrogram Images from EEG signals using Short-Time Fourier transform	CNN + LSTM	[78]
Estimating effective connectivity within the brain’s default mode network (DMN) using the PDC algorithm	3D-CNN	[79]
Estimation of wavelet coherence (WCOH) between DMN regions of the brain using EEG signals	2D-CNN	[80]
Using the projection method to construct EEG signals	CNN	[81]
Power spectral density (PSD) features were extracted for four bands: δ, θ, α, and β	GTSAN	[82]
Build two similarity metric views	MLP	[83]
A MultiView (MV) Feature Extractor	MV-SDGC-RAFFNet	[84]
Automatic generation of brain functional connectivity included in the adjacency matrix and congregating of spatio-temporal features	AMGCN-L	[85]
Constructing brain functional connectivity matrices using a fusion feature called P-MSWC	Lightweight-CNN	[86]
Using multi-scale saliency-encoded spectrogram	TSUnet-CC	[87]
Designing a forward diffusion noise training module to extract noise-independent features	DiffMDD	[88]

DAN: Deep Adaptation Network, LSTM: long short-term memory, GNN: graph neural network, GTSAN: Gated Temporal-Separable Attention Network, MLP: Multi-Layer Perceptron, MV-SDGC-RAFFNet: Multiview Sparse Dynamic Graph Convolution-based Region-Attention Feature Fusion Network, AMGCN-L: Adaptive Multi-time-window Graph Convolutional Network with long-short-term memory, TSUnet-CC: Temporal Spectrogram Unet embedding Cross Channel-wise attention mechanism, and DiffMDD: a diffusion-based deep learning framework for MDD diagnosis using EEG.

**Table 8:** Feature selection methods used in EEG-based depression diagnostic studies.

Feature Selection/Dimensionality Reduction Method	Study
Minimal redundancy maximal relevance (MRMR)	[38,56]
Rank-based feature selection method	[39]
GA	[40,55]
Feature averaging strategy across epochs	[41]
Univariate feature ranking using F-tests	[42]
ReliefF	[42,56]
A two-stage feature selection method named PAR	[43]
Node2vec algorithm	[44,45]
Analysis of Variance (ANOVA) test and correlation analysis	[46]
Fisher score-based feature selection method	[47]
SVM-RFE, LASSO-LR, and PCA	[49]
Sequential backward feature selection (SBFS)	[52]
Synchronization likelihood method	[53]
Neighborhood Component Analysis (NCA)	[54,59,62]
Maximum Likelihood Function (MLE)	[56]
Linear combination and concatenation	[57]
Student’s *t*-test, Wilcoxon test	[58]
Correlation-based feature selection (CFS)	[63]

SVM-RFE: Support Vector Machine-Recursive Feature Elimination and LASSO-LR: Least Absolute Shrinkage and Selection Operator-Logistic Regression.

**Table 9:** Performance of various algorithms in depression diagnosis studies based on TML.

Algorithms	Validation Methods (Training/Testing/Validation)	Top-Performing Classifier	Accuracy (%)	Study
KNN, SVM, CT, ANN	10-fold CV	KNN	76.83	[38]
LR, NB, SVM	10-fold CV (90%/10%/-)	SVM	98	[39]
KNN, KNN-GA, RF, RF-GA, LDA, LDA-GA, CART, CART-GA	LOPOCV	KNN-GA	94	[40]
KNN, LDA, SVM, CK-SVM	5-fold CV (70%/30%/-)	CK-SVM	84.16	[41]
SVM, LDA, NB, KNN, DT	10-fold CV	DT	95	[42]
LR, SVM, LNR	5-fold CV	SVM	98.95	[43]
KNN, SVM, LR, LDA, XGBoost, DT	10-fold CV (90%/-/10%)	KNN	93.3	[44]
KNN, SVM, LR, LDA, XGBoost, DT	10-fold CV (90%/-/10%)	KNN	96	[45]
KNN, NB, DT, MLP, SVM, XGBoost, RF	10-fold CV (53.6%/33%/13.4%)	XGBoost	87	[46]
SVM	LOOCV	SVM	83.67	[47]
SVM (Linear, Quadratic, Cubic, Gaussian radial basis), KNN (Fine, Medium, Coarse, Cosine, Cubic, Weighted)	5-fold CV (70%/30%/-)	Fine KNN	98.43	[48]
DT, SVM, GBDT, NB, KNN	10-fold CV (90%/10%/-)	KNN	88.2	[49]
SVM (Linear, Radial basis function), RF, LR, DT, GB, NB, RB	10-fold CV (90%/10%/-)	RBF-SVM	99	[52]
LC-KSVD, CLC-KSVD	10-fold CV (90%/10%/-)	LC-KSVD	99	[53]
Weighted KNN, SVM (Quadratic)	Hold-out CV (80%/20%/-)	Weighted KNN	99.11	[54]
E-KNN, SVM, MLP	10-fold CV (90%/-/10%)	E-KNN	98.44	[55]
SVM, DT, NB, LDA, LR, Bagging	10-fold CV	SVM	95.23	[56]
SVM, KNN, DT	10-fold CV (90%/10%/-)	SVM	94.03	[57]
SVM, LS-SVM, KNN, DT, RF, Gradient Boosting, (Bagged, Boosted, Stacked) Ensemble	10-fold CV, LOOCV (90%/10%/-)	Stacked Ensemble	99.11	[58]
SVM (Coarse Gaussian, Cubic, Fine Gaussian, Linear, Quadratic, Medium Gaussian), KNN (Coarse, Cosine, Cubic, Fine, Ensemble Subspace, Medium, Weighted), (Coarse, Fine, Ensemble Bagged, Ensemble Boosted) Tree, (Linear, Ensemble Subspace) Discriminant, (Trilayered, Narrow, Wide, Bilayered) Neural Network, Kernel Naïve Bayes (KNB), Logistic Regression Kernel	10-fold CV (75%/25%/-)	KNB	91.8	[59]
KNN	LOOCV, 10-fold CV	KNN	100	[62]
BF-Tree, KNN, AdaBoost	10-fold CV, Nested CV (90%/10%/-)	BF-Tree	96.36	[63]

CT: Classification Tree, ANN: Artificial Neural Network, NB: Naïve Bayesian, KNN-GA: K-nearest neighbor-genetic algorithm, RF-GA: Random Forest-genetic algorithm, LDA-GA: Linear Discriminant Analysis-genetic algorithm, CART: Classification and Regression Tree, CART-GA: Classification and Regression Tree-genetic algorithm, CK-SVM: Conformal Kernel-Support Vector Machine, LNR: Linear Regression, GBDT: gradient-boosting Decision Tree, RBF-SVM: Radial Basis Function kernel-Support Vector Machine, LC-KSVD: Label Consistent K-SVD, CLC-KSVD: Correlation-based Label Consistent K-SVD, E-KNN: enhanced K-nearest neighbor, LOPOCV: Leave-One-Participant-Out Cross-Validation, and LOOCV: Leave-One-Out Cross-Validation.

**Table 10:** Performance of various algorithms in depression diagnosis studies based on DL.

Algorithms	Validation Methods (Training/Testing/Validation)	Top-Performing Classifier	Accuracy (%)	Study
CNN, RF, LSTM, Bi-LSTM, XGBoost, RNN, GRU, Gradient Boosting	10-fold CV (70%/30%/-)	CNN	98.13	[36]
CNN	10-fold CV (90%/10%/-)	CNN	95.49	[64]
DeprNet, AchLSTM, AchCNN, T-LSTM, H-KNN1, H-KNN2, S-EMD, S-SVM, H-DBN	10-fold CV (53.3%/33.3%/13.4%)	DeprNet	99.37	[65]
CNN-LSTM	10-fold CV (70%/15%/15%)	CNN-LSTM	99.12	[66]
2DCNN-LSTM, SVM, KNN, DT	LOOCV	2DCNN-LSTM	95.1	[67]
InceptionTime	10-fold CV (20.9%/66.9%/12.2%)	InceptionTime	91.67	[68]
EEGNet, DeepConvNet, ShallowConvNet	(70%/10%/20%)	EEGNet	94.27	[69]
LSDD-EEGNet, RF, XGBoost, LightGBM, KNN, EEGNet, DeepConvNet	(70%/30%/-)	LSDD-EEGNet	94.69	[70]
CNN, CNN-LSTM, DeprNet, Two-branch CNN	LOSOCV, 10-fold CV	Two-branch CNN	98.45	[71]
HybridEEGNet, SynEEGNet, RegEEGNet, DeepConvNet, AchCNN, EEGNet	10-fold CV	HybridEEGNet	79.08	[72]
MRCNN-RSE, MRCNN-LSTM, EEGNet, DeprNet, 1DCNN-LSTM, 2DCNN-LSTM	5-fold CV	MRCNN-RSE	98.48	[73]
CNN, SVM, KNN	10-fold CV	CNN	94.13	[35]
DAN, DANN, DeepCoral	(87.5%/12.5%/-), (50%/50%/-)	DAN	77	[74]
LDA, SVM, 2DCNN-LSTM, Dictionary Learning	10-fold CV, LOOCV	2DCNN-LSTM	96.33	[75]
MGGCN, SVM, GICN	5-fold CV (70%/30%/-)	MGGCN	99.69	[76]
2D-CNN	5-fold CV (80%/20%/-)	2D-CNN	98.85	[77]
VGG16, AlexNet, Inception, ResNet50, CNN, CNN-GRU, CNN-LSTM	10-fold CV	CNN-LSTM	99.9	[78]
3D-CNN	10-fold CV, 15-fold CV (50%/50%/-)	3D-CNN	95.65	[79]
2D-CNN	10-fold CV (50%/50%/-)	2D-CNN	100	[80]
TCN, LSTM, CNN	8-fold CV	CNN	77.20	[81]
GTSAN, SVM, KNN, LSTM, MLP, GRU, TCN	(80%/20%/-)	GTSAN	98.33	[82]
MLP, SVM, LR, GCN, GAT	10-fold CV (80%/10%/10%)	MLP	99.19	[83]
MV-SDGC-RAFFNet, EEGNet, Shallow ConvNet, Deep ConvNet, TS-SEFFNet, 4D-CRNN, DBGC-ATFFNet	10-fold CV	MV-SDGC-RAFFNet	99.19	[84]
SVM, KNN, FCN, AMGCN, LSTM, CNN-LSTM, ASTGCN, AMGCN-L, GICN, DepHNN, LSDD_EEGnet	10/5-fold CV	AMGCN-L	90.57	[85]
Lightweight-CNN, LDA, LR, SVM, RF, AdaBoost	(70%/30%/-)	Lightweight-CNN	99.87	[86]
TSUnet-CC	LOSOCV	TSUnet-CC	99.22	[87]
DiffMDD, LR, SVM, XGBoost, 1D-CNN-Transformer, CWT-1D-CNN, CWP2D-CNN, EEGNet, InceptionNet, TSception, GRU-Conv, DeprNet, GC-GRU	10-fold CV	DiffMDD	94.0	[88]

Bi-LSTM: Bidirectional long short-term memory, GRU: Gate Recurrent Unit, TCN: Temporal Convolutional Networks, LightGBM: Light Gradient Boosting Machine, DANN: Domain Adversarial neural network, MGGCN: Multi-Granularity Graph Convolution Network, GICN: Graph Input layer attention Convolutional Network, GAT: Graph Attention Network, FCN: Fully connected network, AMGCN: Adaptive Multi-time-window Graph Convolutional Network, and LOSOCV: Leave-One-Subject-Out Cross-Validation.

## Data Availability

Data are contained within the article.
